# Terrestrial snail-mucus mediated green synthesis of silver nanoparticles and in vitro investigations on their antimicrobial and anticancer activities

**DOI:** 10.1038/s41598-021-92478-4

**Published:** 2021-06-22

**Authors:** Pramod C. Mane, Shabnam A. R. Sayyed, Deepali D. Kadam, Manish D.Shinde, Amanullah Fatehmulla, Abdullah M. Aldhafiri, Eman A. Alghamdi, Dinesh P. Amalnerkar, Ravindra D. Chaudhari

**Affiliations:** 1 P. G. Department of Zoology and Research Centre, Shri Shiv Chhatrapati College of Arts, Commerce and Science, Junnar, Pune, 410 502 India; 2 Centre for Materials for Electronics Technology, Panchawati, Off-Pashan Road, Pune, 411008 India; 3 Department of Physics and Astronomy College of Science, King Saud University, P. O. Box 2455, Riyadh, 11451 Saudi Arabia; 4 Department of Technology, Savitribai Phule Pune University, Pune, 411 008 India

**Keywords:** Cancer, Microbiology, Zoology, Materials science, Nanoscience and technology

## Abstract

Over the past few years, biogenic methods for designing silver nanocomposites are in limelight due to their ability to generate semi-healthcare and para-pharmaceutical consumer goods. The present study reports the eco-friendly synthesis of silver nanoparticles from the hitherto unexplored mucus of territorial snail *Achatina fulica* by the facile, clean and easily scalable method. The detailed characterization of the resultant samples by UV–Visible Spectroscopy, FESEM-EDS, XRD and FTIR Spectroscopy techniques corroborated the formation of silver nanoparticles in snail mucus matrix. The resultant samples were tested against a broad range of Gram positive and Gram negative bacteria like *Escherichia coli*, *Staphylococcus aureus*, *Klebsiella pneumoniae*, *Pseudomonas aeruginosa* and a fungal strain *Aspergillus fumigatus* by well diffusion method. The results indicate that silver nanoparticles in mucus matrix exhibit strong antibacterial as well as antifungal activity. The pertinent experiments were also performed to determine the inhibitory concentration against both bacterial and fungal strains. Anticancer activity was executed by in vitro method using cervical cancer cell lines. Curiously, our biogenically synthesized Ag nanoparticles in biocompatible mucus revealed anticancer activity and demonstrated more than 15% inhibition of Hela cells. We suggest an interesting possibility of formulating antimicrobial and possibly anticancer creams/gels for topical applications in skin ailments.

## Introduction

Owing to their intrinsic antimicrobial properties, nanoparticles of oligodynamic noble metals (viz., gold and silver) are on the verge of making unique niche in the fast-moving semi-healthcare and para-pharmaceutical consumer goods such as shampoos, detergents, soaps, cosmetic products, tooth-pastes etc^1^. The customary chemical and physical methods of generating such nano-metallic particles suffer from disadvantages like low yield, high energy consumption, high capital investment, contamination due to solvents, lack of uniform distribution and hindrance due to synthetic additives, capping agents or stabilizing media especially while exhibiting antimicrobial effect^2^. Consequently, sustained efforts are being made to develop clean, green and eco-friendly processes for synthesizing metallic nanoparticles in industrially viable setting. In this context, micro-organisms and plant-mediated biogenic synthesis of metallic nanoparticles appeared to gain immense popularity. The major advantage of using biological materials is the availability of secondary metabolites, amino acids, proteins which are routinely used in the synthetic steps of nanoparticles^3,4^. In fact, microorganisms such as bacteria, fungi, actinomycetes and yeasts have been reported to possess inherent potential to generate nanoparticles either by intra or extra cellular process and are considered as potential micro-factories for nanoparticles generation^1^. On the other hand, plant-mediated phytogenic synthesis of nanoparticles is rapid, cost-effective, easily scalable to bulk-production and free from complex and multiple processing steps like microbial isolation, culturing and maintenance etc. Additionally, specific medicinal properties of certain plants used in phytogenic synthesis can be synergistically beneficial in the therapeutic applications of the resultant nanoparticulate biocomposites. However, biogenic methodologies hitherto reported for the synthesis of nanoparticles are mainly confined to only micro-organisms and plant extracts and use of external bodily secretions of live animals has been hardly reported in eco-friendly biogenic synthesis of metallic nanoparticles involving reduction and stabilization steps^5^. Due to indiscriminate and uncontrolled use of many plant species, the biodiversity is extremely hampered, as many plant species are now on the verge of extinction from the nature. Use of live organisms to obtain their body secretions is far more advantageous over the use of plants, as the organism is not sacrificed and safely returned to the nature thus resulting in biodiversity conservation as well. In the present endeavor, we explored snail mucus externally secreted by *Achatina fulica* in the biogenic synthesis of silver nanoparticles.

*Achatina fulica* is a terrestrial snail, belonging to phylum Mollusca, having a status of “serious agricultural pest” in India. It secretes a sticky, complex, viscous secretion through specialized goblet cells in the columnar epithelium. The mucus serves various functions such as lubrication for the passage of objects, maintains hydrated layer on the epithelium, blocks the pathogens etc. throughout the life cycle of *A. fulica*^6^. Snail mucus is mainly a mixture of glycoproteins, hyaluronic acid and glycolic acid, all of which have long-documented benefits for the skin. The gel like nature of mucus is due to the presence of glycoproteins^7^. Hyaluronic acid in the mucus has moisturizing properties while glycolic acid helps to stimulate collagen responsible for skin-glow and radiant complexion. On account of all such features, snail mucus is an important ingredient of several cosmetic and para-pharmaceutical products^8^. Aside from such cosmetic aspects, it is felt that main ingredients of snail mucus (proteins, amino acids etc.) can contribute/help assist in bio-reduction and bio-stabilization/surface functionalization steps involved in metallic nanoparticles synthesis.

Amongst oligodynamic noble metals, silver nanoparticles find notable applications in molecular diagnostics, in antimicrobial and anti-inflammatory therapies, as well as in devices that are used in several medical procedures^9,10^. Nosocomial infections as well as community-acquired infections caused by multidrug-resistant (MDR) pathogens are recognized as one of the most serious threats in public health settings. To tackle the problem of MDR diseases, novel strategies are being continuously evolved for the drug development with focus on long-term and effective therapies^11^. In this context, it may be noted that the nanomaterial-based drug development approaches cannot exert evolutionary pressure on bacteria and hence can be beneficial in combating MDR if biocompatibility and cytotoxicity issues are addressed aptly^12^. Interestingly, in our previous study, it was observed that the biologically synthesized silver nanocomposites do not exhibit toxicity against non-target organisms^13^. Silver nanoparticles are also used in water filters to kill the pathogenic micro-organisms which cause water borne diseases such as diarrhea^14,15^. In addition to promising antimicrobial potential, nanoparticles have demonstrated effective action against malarial parasites^16–19^.

By bearing in mind the salient aspects of hitherto unexplored snail mucus as novel biomaterial and taking into account the antimicrobial merits of silver nanoparticles, we have carried out the present biogenic synthesis of silver nanoparticles in snail mucus matrix. Besides antimicrobial investigations; we have also performed preliminary in vitro investigations on the anticancer activity of the resultant bio-nanocomposite. It is believed that our research outcomes can add new insights into the science of organic–inorganic hybrid nanomaterials based emergent drug formulations.

## Results and discussion

### Protein estimation

**Figure 1:**
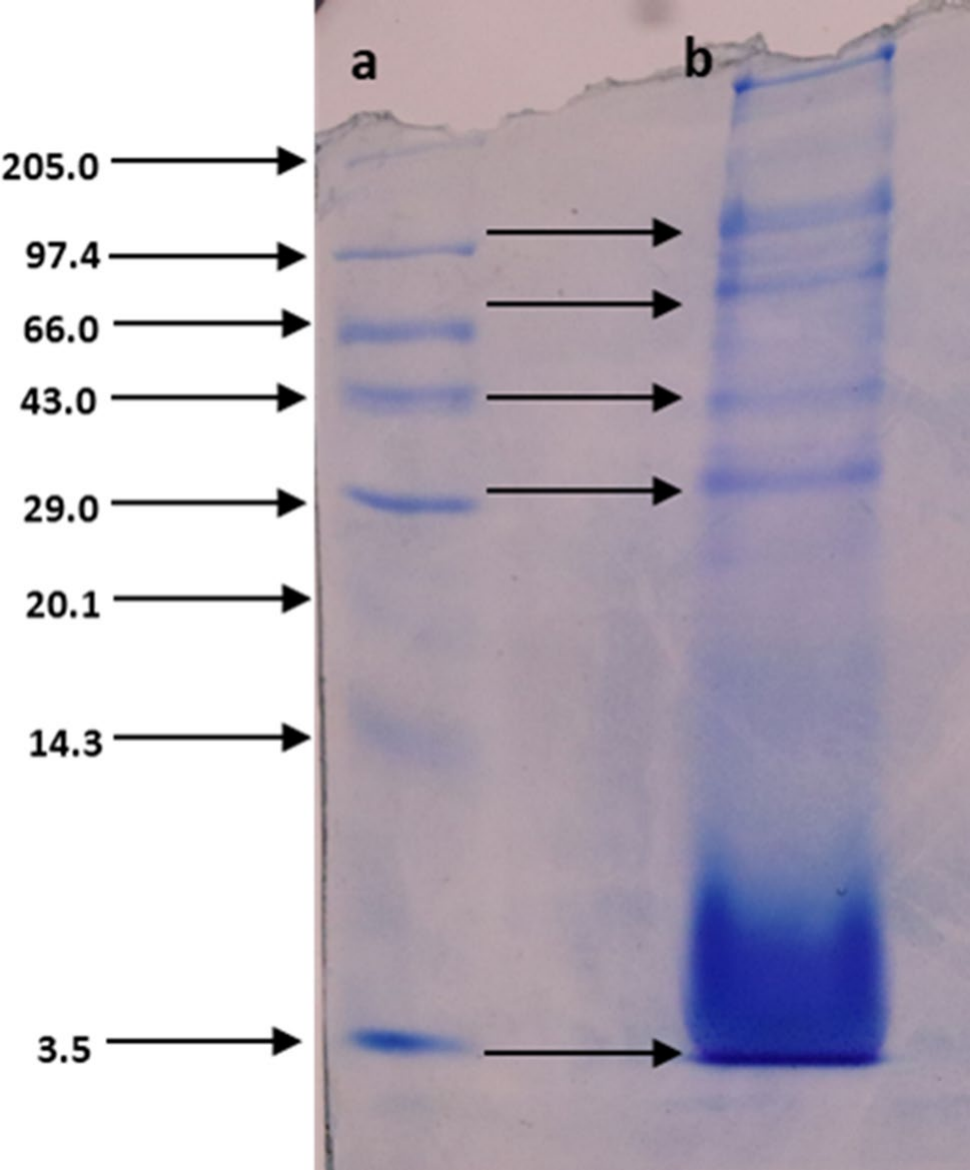
Electrophoretic pattern of mucus (**a**) standard protein marker, (**b**) proteins of *A. fulica* mucus. (Full-length image of the gel is presented in Supplementary Fig. S1).

The mucus was collected and estimation of protein and amino acid contents was performed. The results revealed that the mucus contains 0.463 µg/ml of proteins and 200 µg/ml of free amino acids. To determine the electrophoretic profile of the mucus, it was subjected to SDS – PAGE. The patterns of mucus proteins were verified by the bands clearly seen on the gel. It was observed that the mucus contains proteins of molecular weights 3.5, 14.3, 20.1, 29.0, 43.0, 66.0 and 97.4 kDa (Fig. 1).

Many animals including invertebrates secrete mucus which contains lectins, lysozymes, immunoglobulin, C – reactive proteins, antimicrobial peptides, proteins etc. which are mainly related to immune factors^20^. The proteins present in mucus are involved in immune and stress responses^21^. The mucus is watery, thin fluid containing total soluble proteins, carbohydrates, lipids, amino acids etc^22^. The mucus of *Actinia equina* contains around 24.2% of proteins. The electrophoretic analysis of *A. equine* exhibited fourteen major protein bands, ranging from 12 to 200 kDa^23^. Many researchers recorded proteins from fishes as well. It was also noted that the bands of *Mastacembelus armatus* mucus were observed at 34 kDa, 45 kDa and 144 kDa^24,25^. The present study confirms that the *A. fulica* mucus contains proteins of molecular weights 3.5, 14.3, 20.1, 29.0, 43.0, 66.0 and 97.4 kDa. It was also observed that crude purified extracts from common garden snail, *Helix aspersa muller* mucus (Helix Complex) can actively encourage cell migration, wound healing process, skin protection and antimicrobial activity^8,26^. The snail mucus and 5% chitosan can be used for galenic preparations of anti-inflammatory creams which could be effectively applied for wound healing^27^.

**Figure 2:**
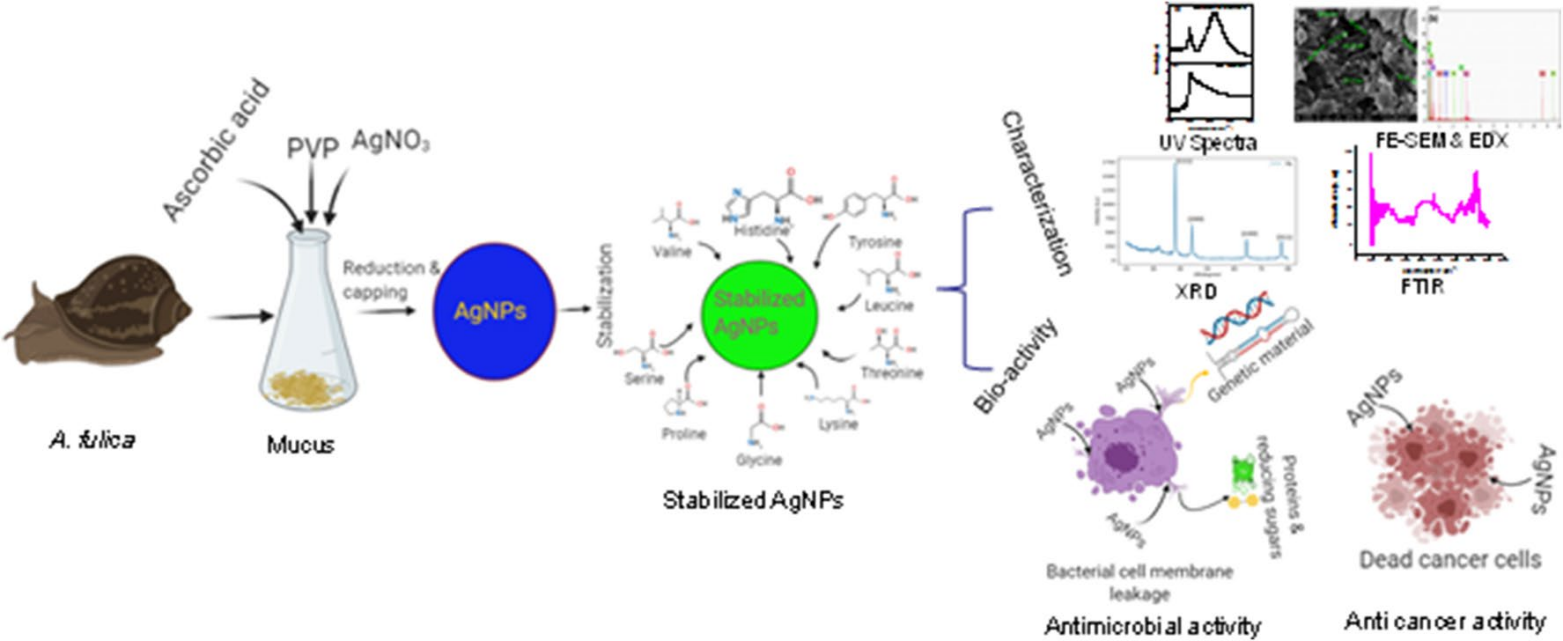
Schematic representation for the generation of silver nanoparticles in snail mucus from *A. fulica* and bioactivity of the resultant bionanocomposite (AgNPs-SM).

Thus, mucus of *A. fulica*, which is rich in different proteins and amino acids, was used in this study for different purposes including synthesis of silver nanoparticles and assessing their antimicrobial and anticancer performance in the composite form (Fig. 2).

### UV–Visible Spectroscopy of *A. fulica* mucus and AgNPs-SM samples

**Figure 3:**
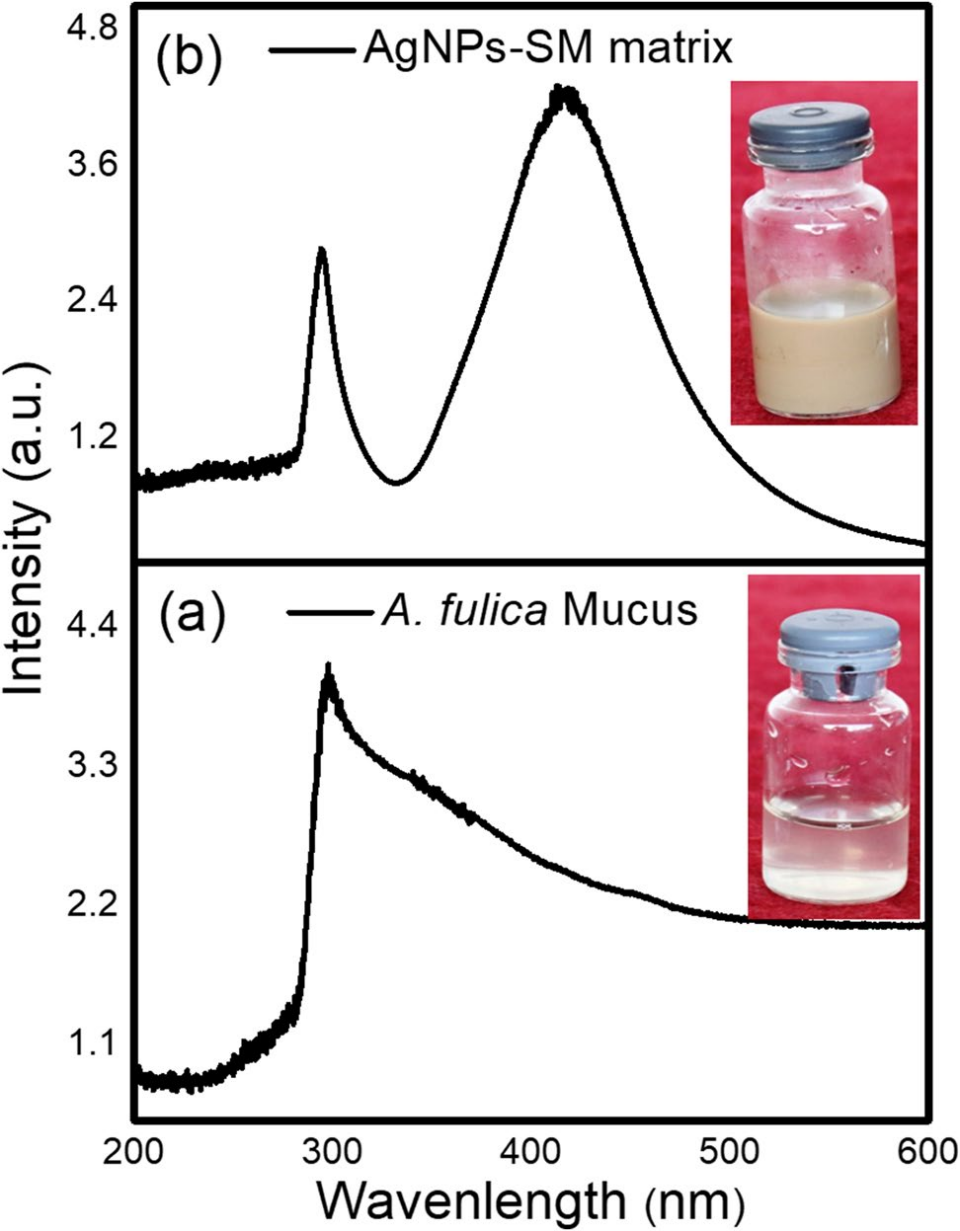
UV–Visible spectra of (**a**) *A. fulica* mucus (**b**) Silver nanoparticles in snail mucus matrix (AgNPs-SM).

The *A. fulica* mucus and silver nanoparticles synthesized in *A. fulica* mucus matrix were studied for their optical properties. The UV–Visible spectra recorded for *A. fulica* mucus and AgNPs-SM samples are displayed in Fig. 3a,b. It is noted that the snail mucus exhibits maximum absorption at 293 nm (Fig. 3a); while the composite spectrum of AgNPs-SM sample (Fig. 3 b) reveals (i) contribution from snail mucus with absorption peak centered at 293 nm and (ii) relatively sharp absorption peak centered at 420 nm attributable to characteristic Surface Plasmon Resonance (SPR) phenomenon reported for spherical silver nanoparticles^13^. The noticeable contribution from snail mucus and presence of relatively sharp characteristic SPR peak in the spectrum evidently suggests key role of mucus as bio-stabilizing medium in controlling the spontaneous aggregation of Ag nanoparticles. Furthermore, we have observed for any visual change in color for various dispersion admixtures of the individual reactants viz*.,* (a) PVP–silver nitrate, (b) ascorbic acid–silver nitrate, (c) PVP–ascorbic acid–silver nitrate and (d) snail mucus–PVP–ascorbic acid and also recorded their UV–Visible spectra (See Supplementary Fig. S2 and Supplementary Fig. S3 a, b, c & d). From such study, we could observe only one absorption peak around 295 nm for all the four admixtures. This observed absorption peak for all the admixtures incidentally matches with that of snail-mucus and might be due to either dominant contribution of the individual reactants in the admixtures or overlapping influence of the maximum absorption peaks of PVP and ascorbic acid around 320 nm and 265 nm, respectively^28–29^. Besides bio-stabilization, snail-mucus contents especially amino acids can facilitate the controlled reduction of silver nitrate to nanoscale silver particles as illustrated in Fig. 2.

The UV spectral analysis of *A. fulica* mucus shows a sharp peak between 200–300 nm. It was reported that the fish mucus exhibits more than one peak in spectral analysis^30^. The absorbance of all types of mucus was around 290 nm which can be ascribed to the presence of nucleic acid and proteins in mucus^31^.

### FESEM with EDS analysis of AgNPs-SM Sample

**Figure 4:**
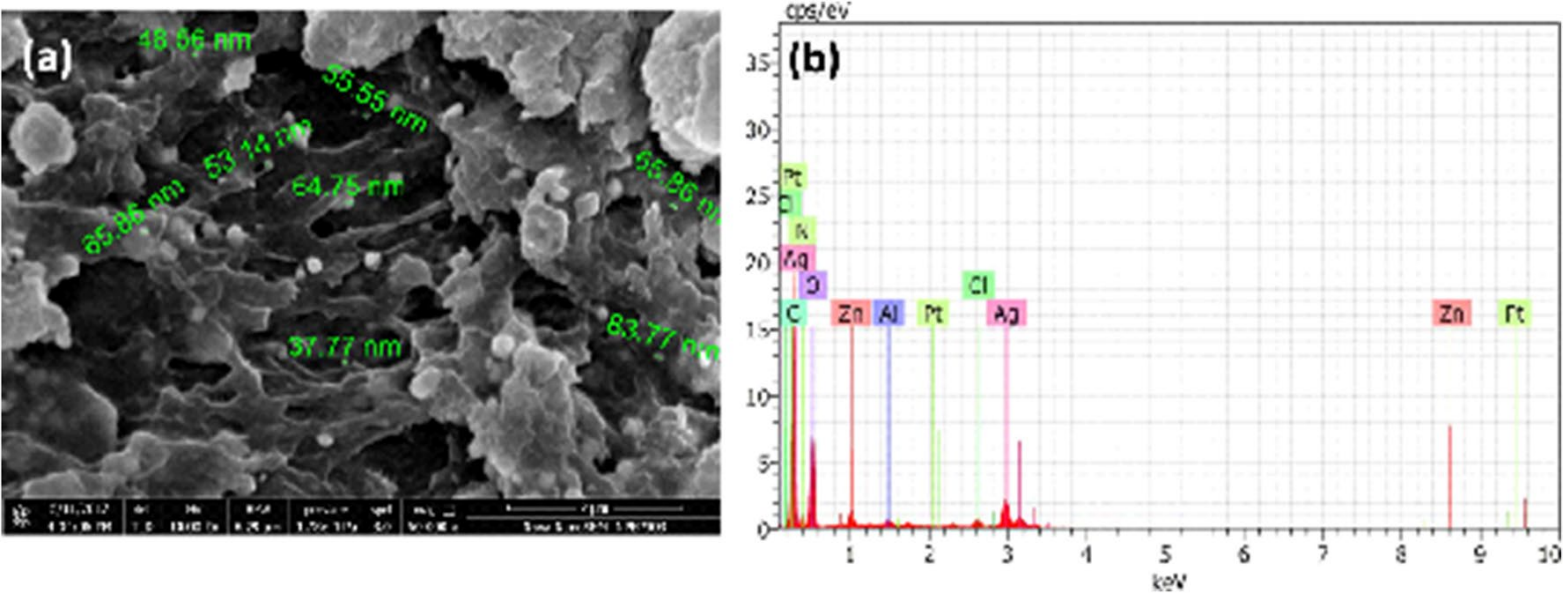
(**a**) FESEM image and (**b**) EDS spectrum of silver nanoparticles synthesized using *A. fulica* mucus.

Figure 4a,b displays FESEM image and EDS spectrum of silver nanoparticles formed in *A. fulica* mucus matrix. Polydispersed and broadly spherical silver nanoparticles exhibiting size in the range of 37 nm – 87 nm were observed on the surface as well as the interior side of biological matrix of *A. fulica*. Thus, FESEM image hints at bio-stabilization of Ag nanoparticles in *A. fulica* mucus matrix in composite form. The pertinent EDS spectrum (Fig. 4b) mainly discloses the presence of silver with other elements namely, zinc, oxygen and chlorine, usually found in typical biological synthesis protocols^32^. Conspicuous absence of calcium is a noteworthy factor in the present analysis.

### XRD Analysis of AgNPs-SM

**Figure 5:**
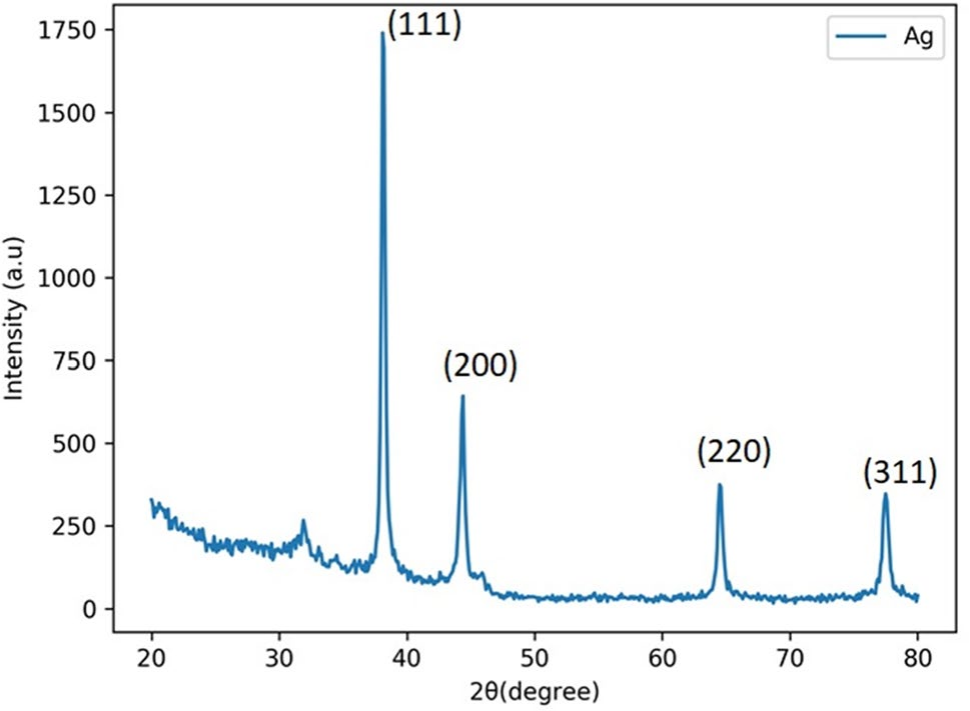
X-ray diffractogram of silver nanoparticles synthesized within *A. fulica* mucus matrix.

Typical X-ray diffractogram of the silver nanoparticles synthesized in *A. fulica* mucus is presented in Fig. 5. It confirms formation of cubic (fcc) silver as evidenced by XRD peaks corresponding to (111), (200), (220) and (311) planes which match well with JCPDS card no. 04-0783 for the cubic silver.

### FTIR Spectroscopic Analysis of AgNPs-SM

**Figure 6:**
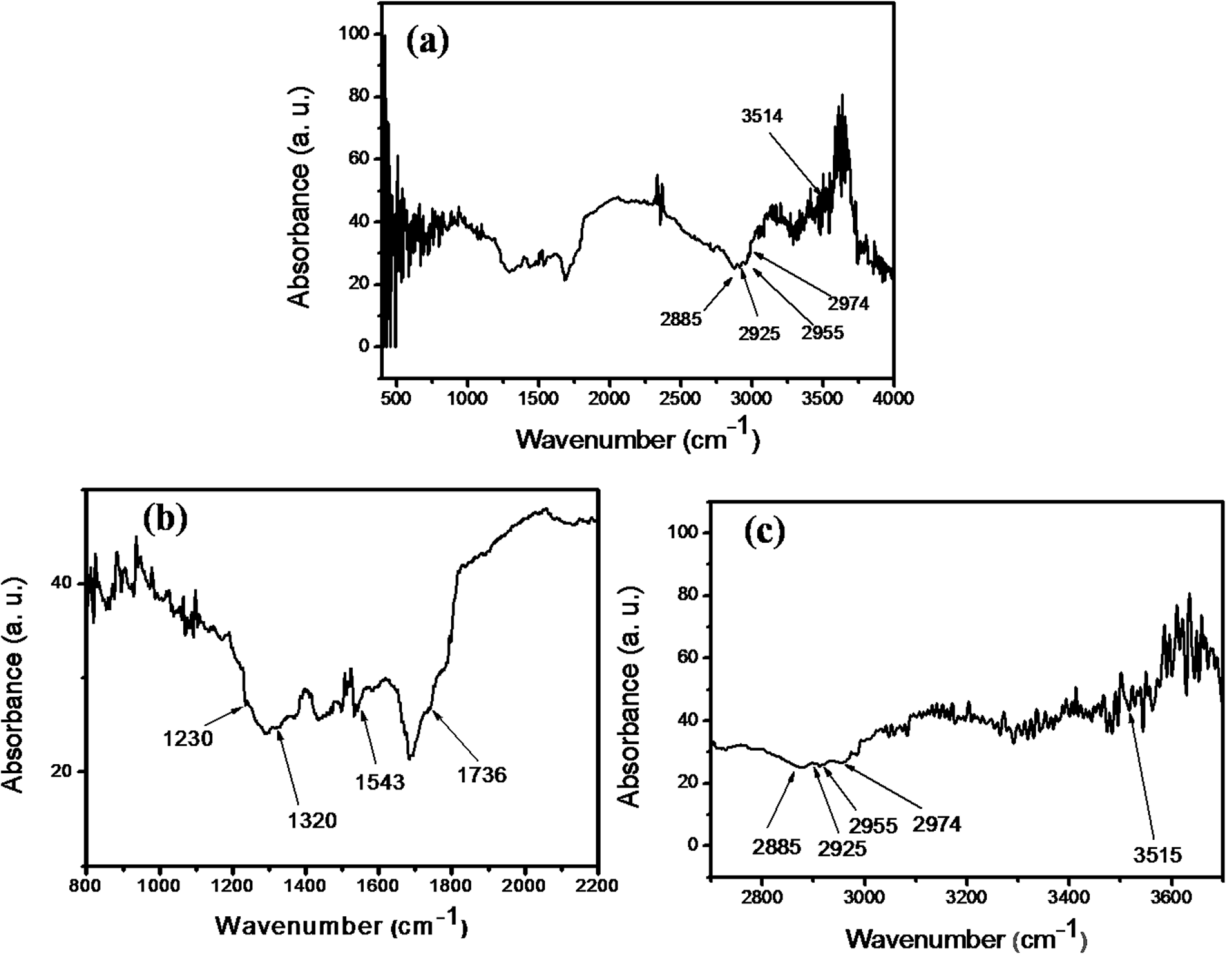
FTIR spectra of AgNPs-SM sample recorded at different spectral regions—(**a**) 400–4000 cm^−1^, (**b**) 800–2200 cm^−1^ and (**c**) 2700–3700 cm^−1^.

FTIR spectral analysis of the typical AgNPs-SM sample was carried out in the range of 400-4000 cm^−1^ (Fig. 6a). The spectral analysis in the narrow range of 800 cm^–1^ to 2200 cm^–1^ is given in Fig. 6b. In this region, the spectral profile indicates the presence of a protein component with β-sheeting and β-turns that are ascribable to the band characteristics of Amide II (1543 cm^–1^)^33^. The band due to Amide I (1643 cm^–1^) is suspiciously absent. Additionally, numerous bands in the region between 900 cm^–1^ and 1450 cm^–1^ have been noted which stand for a combination of bands implying the presence of ionised and unionised carboxylic acid, CH_2_, CH_3_ and –OH as well as secondary amide echoes^26^. Ester bonds observed in some sugars are seen in the form of bands at 1736 cm^–1^ and 1230 cm^–1^. The spectrum obtained in the range of 2700 cm^–1^ to 3700 cm^–1^ (Fig. 6c) revealed the higher wavenumber bands attributable to the presence of CH_2_ around 2855 cm^–1^ and 2925 cm^–1^ probably from a lipid component in the mucus, while CH_3_ echoes (2955 cm^–1^ and 2974 cm^–1^) were also noted. These components are also manifested at lower wavenumbers (1320 cm^–1^, Fig. 6b). The bands observed in the range of 2800 cm^–1^ and 3000 cm^–1^ possibly signify aromatic echoes in addition to exhibiting the presence of bonded OH within the COOH moiety (Fig. 6c). The signal relating to bonded OH was specifically detected at 3515 cm^–1^ and this feature may be due to the presence of sugar side-chains rather than the protein core. Benzene overtones occurring at the low wave numbers (1800 cm^–1^ to 2000 cm^–1^) are also not found.

### Antimicrobial activity

Our biogenically synthesized silver nanoparticulate composites (AgNPs-SM) were assessed for the antimicrobial assay against the Gram negative and Gram positive bacterial strains.

**Figure 7:**
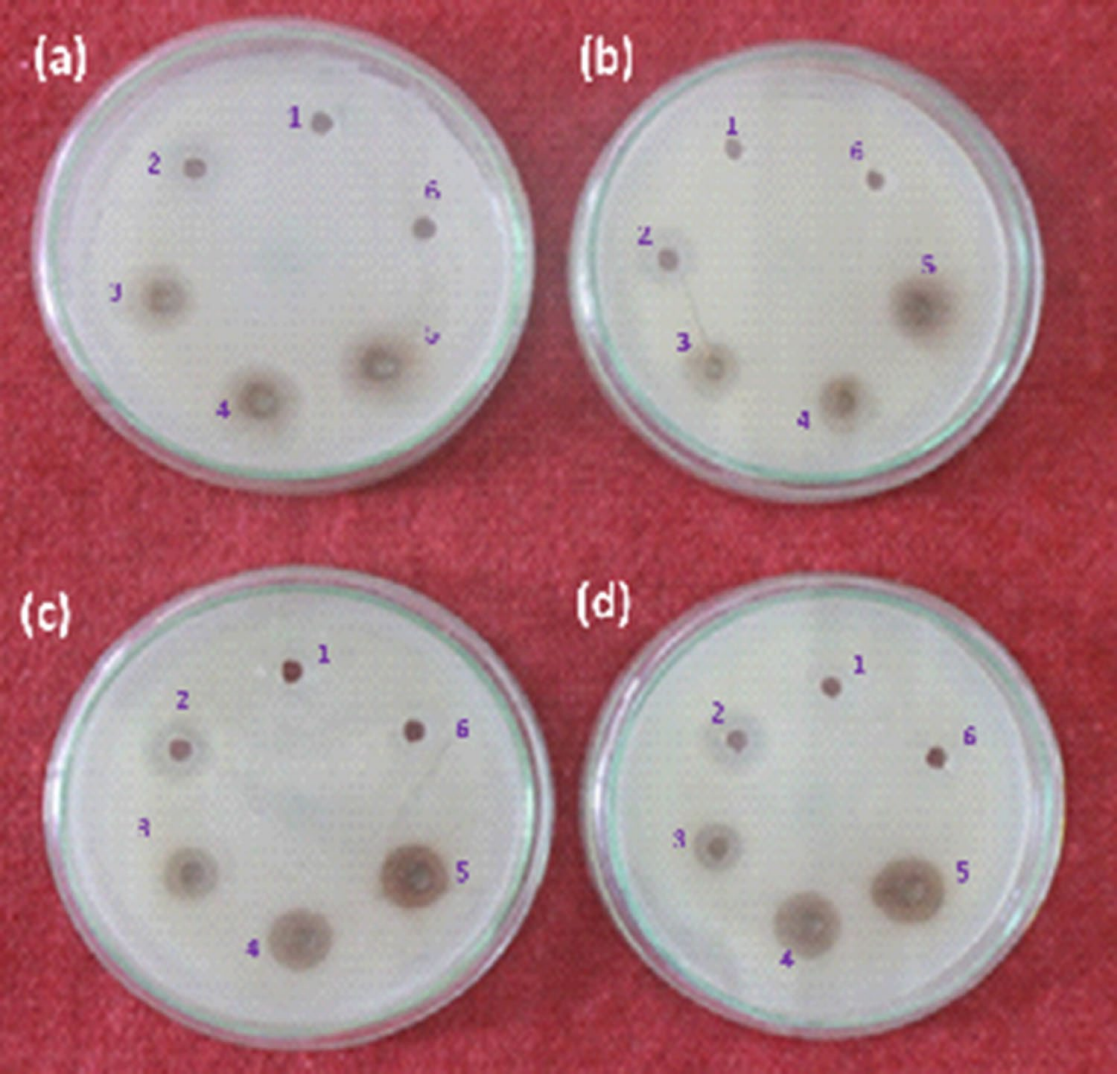
Antimicrobial activity of AgNPs-SM against (**a**) *Klebsiella pneumoniae* (**b**) *Escherichia coli* (**c**) *Pseudomonas aeruginosa* (**d**) *Staphylococcus aureus.* In each plate, (1) AgNO_3_, (2) Ciprofloxacin/ Clotrimazole, (3) 25 µg of Ag NPs, (4) 50 µg of Ag NPs, (5) 75 µg of Ag NPs and (6) *A. fulica* mucus were added, respectively.

The antibacterial activity of the dispersed biogenic silver nanoparticles was verified by observing a clear zone of inhibition (ZI). No zone of inhibition was found in the vehicle control well which suggests that the antimicrobial activity was specifically due to the silver nanoparticles stabilized in mucus matrix. All the selected bacterial strains displayed a noticeable zone of inhibition (Fig. 7). In this study, we also compared the antimicrobial performance of the resultant Ag nanoparticles in mucus matrix with that of standard antibiotic ciprofloxacin. Besides, we have checked the antimicrobial activity of the powdered products formed through the combinative admixtures of individual reactants viz*.,* (a) PVP-ascorbic acid- silver nitrate and (b) ascorbic acid—silver nitrate as controls to highlight the contribution of Ag nanoparticles generated in snail mucus matrix. We could not observe an appreciable antimicrobial activity in case of these products (See Supplementary Fig. S4 a and b). Since remaining admixtures of snail mucus–PVP–ascorbic acid and PVP–silver nitrate did not disclose apparent formation of any powder product, their antimicrobial activity was not carried out. Overall, it has been observed that silver nanoparticles-mucus matrix based nanocomposite dispersion exhibits better antimicrobial activity against *Staphylococcus aureus* (ZI : 16 ± 1.78 mm)*, Pseudomonas aeruginosa* (ZI : 15 ± 2.68 mm) and slightly less against *Klebsiella pneumoniae* (ZI : 14 ± 1.78 mm), *Escherichia coli* (ZI : 14 ± 2.68 mm) and *Aspergillus fumigatus* (ZI : 15 ± 0.89 mm) at 75 µg concentration of nanoparticles.

### Minimum Inhibitory Concentration (MIC), Minimum Bactericidal Concentration (MBC) and Minimum Fungicidal Concentration (MFC) of silver nanoparticles in snail-mucus matrix

**Figure 8:**
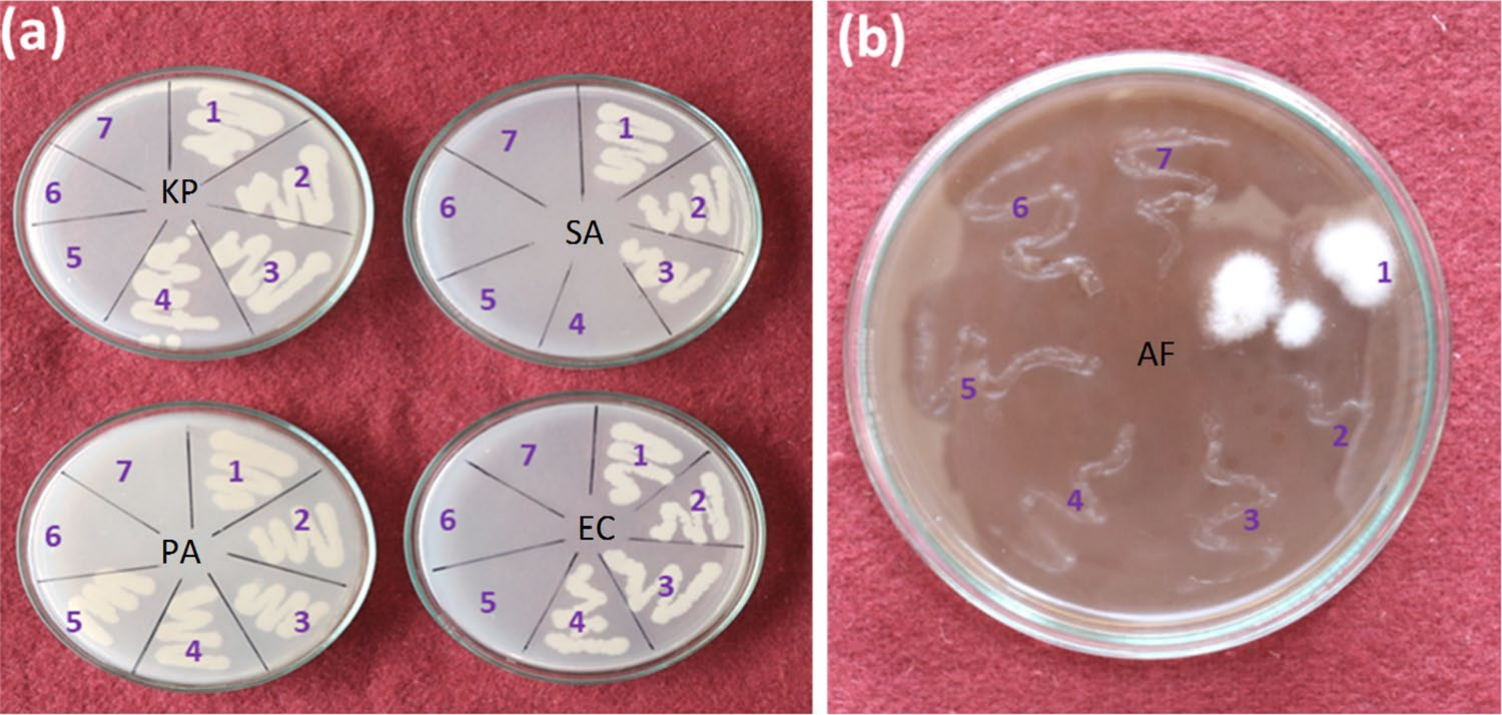
MIC and MBC/MFC of silver nanoparticles (AgNPs) synthesized in *A*. *fulica* mucus matrix. In each plate represents (1) control and AgNPs concentrations of—(2) 2 µg/mL, (3) 2.5 µg/mL, (4) 3 µg/mL, (5) 3.5 µg/mL, (6) 4 µg/mL and (7) 4.5 µg/mL, respectively.

After confirming antimicrobial activity of biogenically synthesized silver nanoparticles through agar well diffusion assay, values of MIC, MBC and MFC were determined for all the selected micro-organisms. The typical experiments were conducted by using 10^6^ CFU/ml of microbial concentrations and various concentrations of silver nanoparticles (2, 2.5, 3, 3.5, 4 and 4.5 μg/ml). It was observed that, after completion of the incubation period, visual growth of *K. pneumoniae, E. coli, P. aeruginosa, S. aureus* and *A. fumigatu*s was not perceived when supplemented with 3.5, 3.5, 4.0, 3.0 and 2.0 μg/ml of silver nanoparticles, respectively (Fig. 8). The corresponding MBC and MFC values are summarized in Table 1.

**Table 1:** Zone of inhibition and MIC of silver nanoparticles synthesized in *A. fulica* mucus matrix.

Name of the microorganisms	Zone of inhibition ( mm )		MBC/MFC (µg/ml)
	AgNPs	Positive Control (Ciprofloxacin/ Clotrimazole)	AgNPs
*Klebsiella pneumoniae*	14 ± 1.78	15 ± 1.78	3.5
*Escherichia coli*	14 ± 2.68	14 ± 1.54	3.5
*Pseudomonas aeruginosa*	15 ± 2.68	15 ± 2.36	4.0
*Staphylococcus aureus*	16 ± 1.78	13 ± 2.36	3.0
*Aspergillus fumigatus*	15 ± 0.89	00	2.0

All the values are mean of three replicates, ± standard deviation.

To overcome the problem of antibiotic resistance in bacteria, use of silver nanoparticles seems to be a good alternative to the present generation antibiotics. The present study explored silver nanoparticles—mucus matrix based bionanocomposite for antimicrobial activity against four bacterial strains and one fungal strain. It is worthwhile to mention that, amongst other nanoparticles, silver nanoparticles had strongest antibacterial activities against several pathogenic bacteria^34^. Herein, the antimicrobial activity of silver nanoparticles—mucus matrix based bionanocomposite is compared with standard antibiotic i.e. Ciprofloxacin/ Clotrimazole. The result of this study indicated that silver nanoparticles—mucus matrix based bionanocomposite exhibited slightly more antimicrobial activity than Ciprofloxacin/ Clotrimazole as shown in Table 1.

In literature, numerous reports are available on green synthesis of metallic nanoparticles by using various biological sources including fungi, bacteria, plants etc. and elucidating their functional superiorities over metallic nanoparticles generated by traditional chemical and physical methods^1–5,11,35^. In the present study, we successfully synthesized the silver nanoparticles in *A. fulica* mucus matrix and ascertained its potential for biological applications including antimicrobial and anti cancer activities. It may be noted that Gubitosa et al.also carried out the synthesis of gold nanoparticles by using mucus secreted by garden snails *Helix aspersa Muller* and used the resultant bionanocomposite for biomedical applications involving potential anti-inflammatory properties^36^. At this juncture, it is felt that the functional combination of (i) broad-spectrum antimicrobial silver nanoparticles and (ii) anti-inflammatory as well as skin rejuvenating ingredients of the snail-mucus in our AgNPs-SM composite can be explored in topical treatment of acne, an extremely common bacterial skin disease. In particular, snail mucus possess dermis hydrating and collagen regulating properties^8,25,36^ which can control formation of acne scars. Additionally, it may be recalled that acne is susceptible to develop resistance for the long-term routine antibiotic treatment which may not be ordinarily anticipated in case of metallic nanoparticles. As a whole, it may be fascinating endeavor to realize AGNPs-SM based topical cream/gel for effective acne treatment as well as for rapid wound healing without leaving scar on the affected skin.

As compared to bacterial diseases, fungi mediated diseases are tedious to control, because currently very few antifungal drugs are available^37^. In view of this scenario, there is an urgent and inevitable need to formulate antifungal agents which must be cost effective, eco-friendly and most importantly biocompatible^38^. Literature survey indicates that, silver nanoparticles play an important role as an antifungal agent^39^. For example, silver nanoparticles exhibited a good antifungal activity against several fungi like *Aspergillus niger, Candida albicans, Phoma glomerata, Fusarium semitectum* etc^40,41^. It was mentioned that, silver nanoparticles not only inhibit human and plant fungi but also impede indoor fungi including *Penicillium brevicompactum* and *Aspergillus fumigates*^27,42^.

### Growth curve study

**Figure 9:**
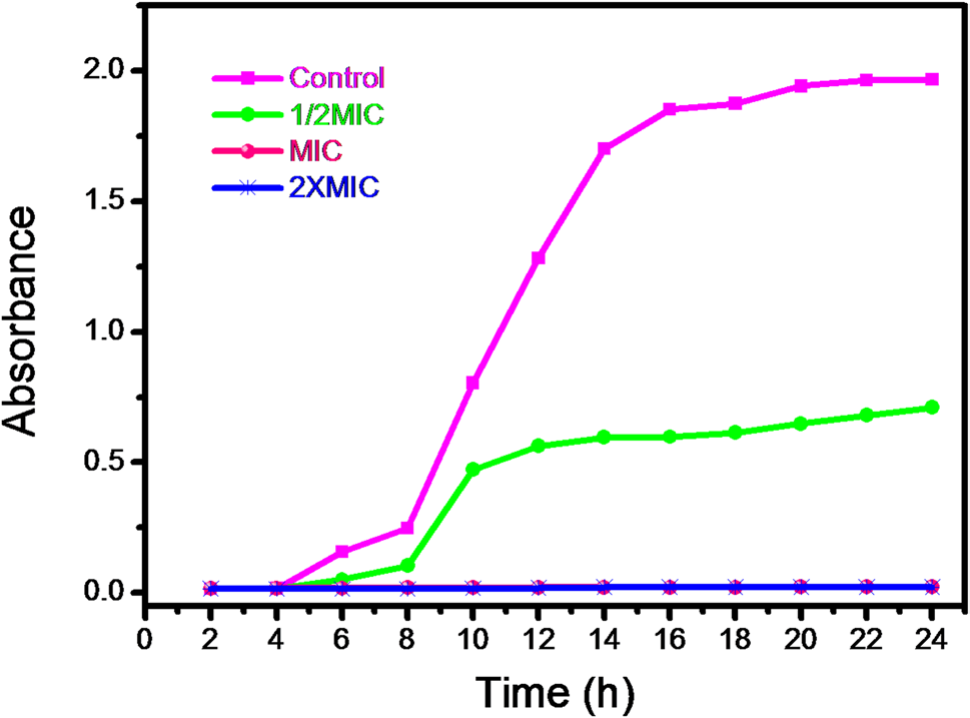
Time kill assay of *S. aureus* treated cells with AgNPs-SM bionanocomposites.

To investigate the effect of silver nanoparticles on growth curve, *S. aureus* was selected as a model bacterial organism, as it exhibited maximum zone of inhibition to the present silver nanoparticles-mucus matrix based dispersion sample. The growth curves of such silver based bionanocomposite treated *S. aureus* cells are shown in Fig. 9. It is seen that the growth curve of *S. aureus* was inhibited at MIC (3.5 μg/ml) and 2 × MIC (7.0 μg/ml) of silver nanoparticles.

The mechanism involved in killing of bacterial cells may be explained on the basis of ROS (Reactive Oxygen Species) generation which inhibits the bacterial growth most effectively on Gram negative strains^43,44^.

### Mechanism of action of silver nanoparticles on *S. aureus* cell membrane

**Figure 10:**
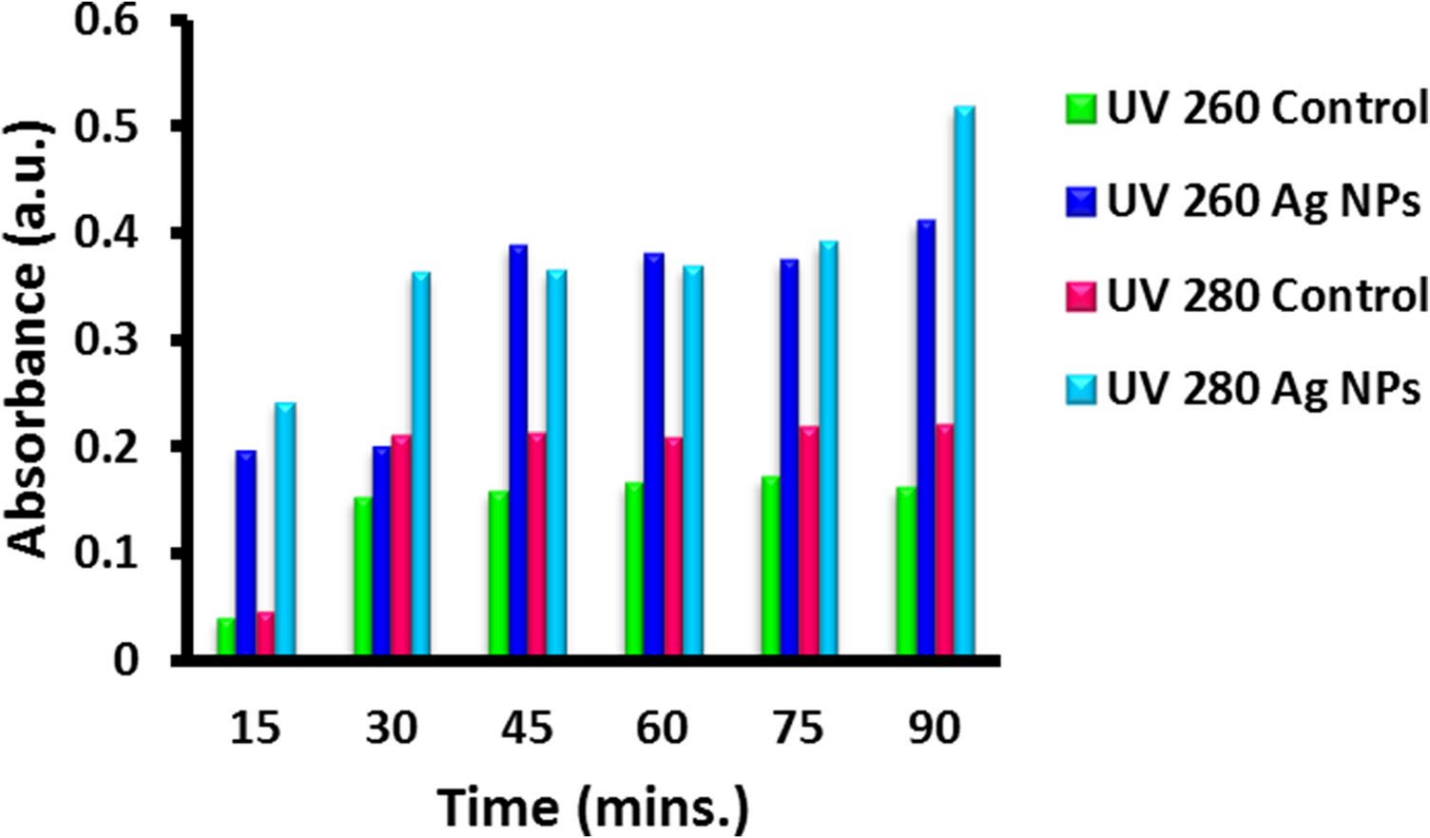
Leakage at UV_260_ and UV_280_ (Heipieper method) of *S. aureus* treated with AgNPs-SM bionanocomposites.

In the present study, it was observed that silver nanoparticles could enhance protein leakage by increasing membrane permeability of *S. aureus*. The leakage at UV-260 and UV-280 absorbing material for the period of 90 min was monitored and the results are summarized in the Fig. 10. The absorbance at 280 nm was increased in 15 min as compared to the absorbance at 260 nm. This suggests that silver nanoparticles alter the membrane permeability of cells which, in turn, results in the leakage of UV- 260 and UV- 280 absorbing materials.

It is the well established experimental fact that silver nanoparticles can exhibit better antimicrobial activity against Gram positive and Gram negative bacteria. In case of *E. coli,* it has been speculated that, silver nanoparticles accumulate in the cell wall and lead to cell death by formation of “pits”^45^.

**Figure 11:**
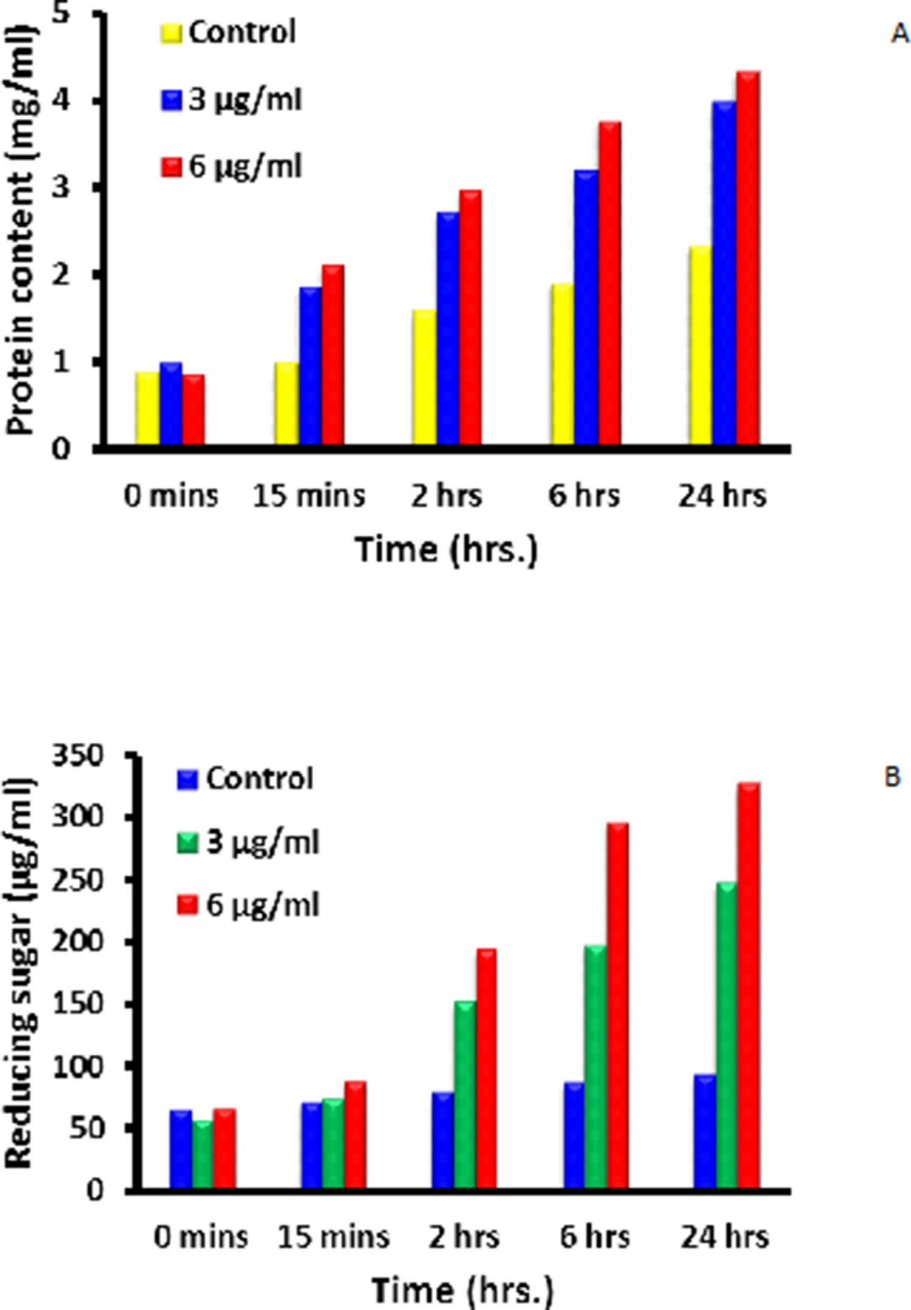
Leakage of (**A**) Proteins & (**B**) Reducing sugars from *S. aureus* after treatment with AgNPs-SM bionanocomposites.

**Figure 12:**
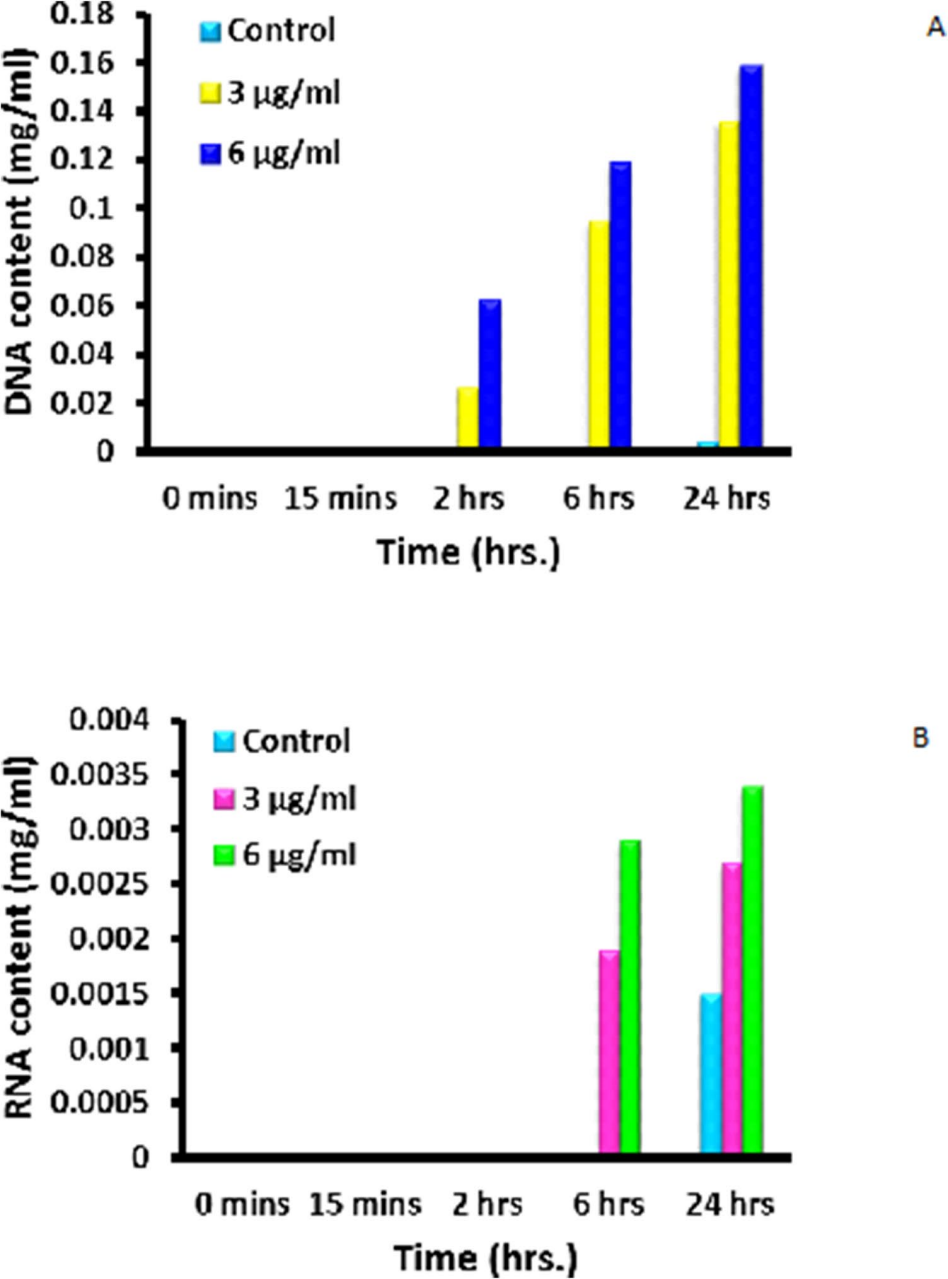
Leakage of (**A**) DNA and (**B**) RNA from *S. aureus* after treatment with AgNPs-SM bionanocomposites.

Herein, we also investigated the effects of as-synthesized silver nanoparticles on leakage of proteins, reducing sugars, DNA and RNA against *S. aureus*. The results obtained are presented in Figs. 11 and 12.

Figure 11A exhibits the protein leakage from *S. aureus* treated with silver nanoparticles and compared with control. From the Fig. 11A, it is evident that, after 24 h of incubation, control set shows 2.32 mg/ml of protein while 3 µg/ml and 6 µg/ml silver nanoparticles treated sets exhibit 3.98 mg/ml and 4.32 mg/ml of protein content, respectively. Whereas Fig. 11B exhibits the reducing sugar leakage from *S. aureus* treated with silver nanoparticles and compared with control. Here it has been realized that, after 24 h of incubation, control set shows 93 µg/ml of reducing sugar while 3 µg/ml and 6 µg/ml silver nanoparticles treated sets produce 247 µg/ml and 327 µg/ml of reducing sugar content, respectively.

Figure 12A indicates the DNA leakage from *S. aureus* bacterial cells treated with silver nanoparticles and compared with control. From the Fig. 12A, it can be observed that, after 24 h of incubation, control set shows 0.0038 mg/ml of DNA while 3 µg/ml and 6 µg/ml silver nanoparticles treated sets exhibit 0.136 mg/ml and 0.159 mg/ml of DNA content, respectively. Figure 12B exhibits the RNA leakage from *S. aureus* bacterial cells treated with silver nanoparticles and compared with control. Here, it can be noted that, after 24 h of incubation, control set shows 0.0015 mg/ml of RNA content while 3 µg/ml and 6 µg/ml silver nanoparticles treated sets exhibit 0.0027 mg/ml and 0.0034 mg/ml of RNA content, respectively.

During the study of membrane leakage, proteins, reducing sugars, DNA and RNA of *S. aureus* as a model organism, the silver nanoparticles may lead to formation of ROS e. g. superoxides and hydroxyl radicals, which presumably lead to disruption of the bacterial cell membrane^46^. It is also reported that, metal nanoparticles increase ROS through reaction of metal ions with thiol group of enzymes and exerts toxic effects related to oxidative stress^47^. When bacterial cell comes in contact with nanoparticles, it inhibits respiratory enzymes leading to ROS generation and thus damaging the bacterial cell^48^. To judge the bioactivity of biogenically generated nanomaterials, several properties of nanoparticles must be studied which are responsible for the resultant efficacy and toxicity. These properties mainly include particle size, its distribution, shape, surface charge, crystalline phase etc. It has been reported that such properties of nanoparticles can greatly influence the biological activities of the biogenically generated nanoparticles/composites^49^.

### Cytotoxicity of Ag NPs

Along with antibacterial and antifungal activities, we have also investigated the anticancer activity of green synthesized silver nanoparticles against HeLa (cervical cancer) cells. The cytotoxicity result specified that AgNPs-SM bionanocomposite showed more than 15 % inhibition of Hela cells. The present material was further selected for the dose response studies. The dose response studies were carried out at different concentrations (100, 30, 10, 3, 1 µg/ml) (Table 2).

**Table 2:** IC50 and IC90 value of Ag NPs.

Concentration of Ag NPs	0	1	3	10	30	100	IC50	IC90
Percent inhibition	0.00	15.59	17.73	19.85	39.26	58.65	64.05	> 100

**Figure 13:**
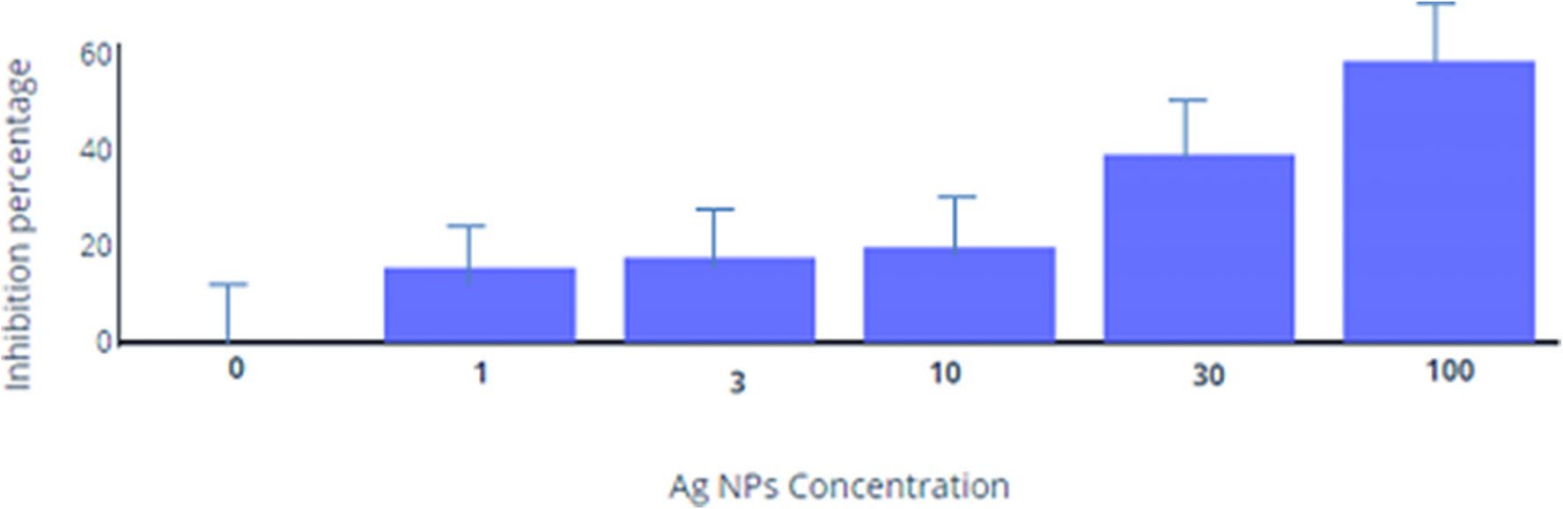
Dose response studies of AgNPs-SM bionanocomposite against HeLa cells.

It was noticed that all the concentrations of Ag NPs were capable of inhibition of HeLa cells in a dose-dependent manner. The inhibition activity increases with concentration in the order of 100>30>10>3>1 (Fig. 13). The highest effective IC50 and IC90 reported for Ag NPs were 64.05 μg/ml and >100, respectively.

Cervical cancer exhibited third leading causes of cancer death after breast and lung cancers in the world, where, almost 90% of cervical deaths occur in developing countries^50,51^. Some researchers observed the cytotoxic effect of silver nanoparticles in HeLa cells^52^. It was recorded that, silver nanoparticles can lead to cell deaths which are concentration dependent. The inhibition activity increases with concentration in the order of 100 > 30 > 10 > 3 > 1 µg/ml. The main advantage of using silver nanoparticles is that it cannot just induce apoptosis but also can recognize the cancer cells^53^. It was witnessed that the combination of anticancer agents with nanoparticles shows significant inhibition in cell viability and proliferation, for instance, combination therapy of CPT and AgNPs has shown significant inhibition in cell viability and proliferation of HeLa cells by modulating cellular signaling molecules associated with cell survival, cytotoxicity, and apoptosis^50^. Further, the synthesized silver nanoparticles with other reported anticancer agents and their derivatives can certainly provide a beneficial effect in the treatment of different types of cancers. It was also noted that, biologically synthesized silver nanoparticles lead to cell death very effectively. Chitosan coated silver nanoparticles exhibited increased cell mortality rate^54^. More importantly, silver nanoparticles which are biologically synthesized demonstrate significant toxicity against MCF7 and T47D cancer cell lines^55^. Silver nanoparticles exhibit target cell-specific toxicity against human lung cells^56^.

## Materials and methods

### Collection of snails and mucus extraction

Live *Achatina fulica* snails were collected from the nearby Northern Western Ghats, rich biodiversity region of Maharashtra, India by the hand picking method. Before collecting the mucus, snails were starved for 24 h to avoid any contamination. Around 50 healthy individuals were selected for mucus extraction and were fed with cabbage or papaya leaves for 2 days. The mucus was extracted from *A. fulica* snails by stimulating its pedal glands. Approximately 5 ml of mucus was collected per two individual snails at a time. The collected mucus (approximately 125 ml) was mixed together and stored at −20 $^\circ$C until being used for further experiments^57^. After the extraction was over, the snails were freed back to their original natural habitat.

### Estimation of proteins, free amino acids and sodium dodecyl sulfate–polyacrylamide gel electrophoresis (SDS-PAGE) of snail mucus

Total proteins were estimated from the mucus sample by the Lowry et al.method^58^. Total free amino acids were estimated by the Ninhydrin method^59^. SDS-PAGE of isolated mucus sample was carried out on a vertical gel electrophoresis system (Hoffer). Proteins were electrophoresed on 12% separating gel overlaid with 5% stacking gel. The protein bands of mucus were compared with standard protein marker (GeNei) with broad range molecular weights (3.5 to 205 kDa). The protein profile was visualized, documented and preserved by the modified method^60^. All the experiments including estimation of proteins, total amino acids etc. were replicated thrice.

### Synthesis of silver nanoparticles using *A. fulica* snail mucus

The biocompatible silver nanoparticles were synthesized within mucus matrix of *A. fulica*. In a typical procedure, diluted (1:9 v/v) *A. fulica* mucus (100 ml) was mixed with 284 mM ascorbic acid and 66 mM polyvinyl pyrrolidone and 35 mM AgNO_3_. The initial pH of the diluted mucus was around 7 but, after the addition of ascorbic acid and polyvinyl pyrrolidone, pH of the resultant mixture was found to be changed to 4. The whole mixture was stirred at 250 rpm for 45 min and exposed to sunlight to sharpen the color of the dispersion mixture from gray to brown within short time period of 2–5 min implying the formation of silver nanoparticles^61^ . This distinctive change in colour can be attributed to size and shape dependent Surface Plasmon Resonance of Ag nanoparticles in solution when exposed to visible light^13^. After visually noticing such a change in the color of the dispersion mixture, the powder product formed was collected by centrifugation at 12,000 RPM for 10 min and washed several times with double distilled water until its pH becomes neutral. Silver nanoparticles-snail mucus nanocomposite powder (referred hereafter as AgNPs-SM) was finally obtained by drying the resultant semi-solid mass in lamellar air flow at room temperature for 1 day. For the sake of comparison, various combinative admixtures corresponding to individual reactants namely snail mucus, silver nitrate, PVP and ascorbic acid were also prepared.

### Physico-chemical characterization of the resultant nanoparticulate composites

*A. fulica* mucus and typical AgNPs-SM samples were separately suspended in sterile distilled water for performing UV–visible spectroscopy study. The spectra were recorded by using UV visible NIR spectrophotometer (JASCO V-770) in the wavelength range of 200—600 nm against distilled water as baseline solution by using optiglass cuvette with path length of 10 mm and bandwidth of 1.0 nm. Surface morphological features and the pertinent elemental composition of AgNPs-SM were determined by acquiring Field Emission Scanning Electron Microscopy (FE-SEM) images using FEI, Nova NanoSEM NPEP303. For this purpose, the synthesized AgNPs-SM powder was directly sprinkled on the conducting carbon tape attached to aluminum stub. It was then coated with very thin conducting gold film to minimize the effects arising due to charging. Silver nanoparticles-snail mucus nanocomposite powder (AgNPs-SM) obtained by drying the resultant semi-solid mass in lamellar air flow at room temperature was used for the structural analysis by X-ray diffractometry (XRD). XRD pattern of the dried AgNPs-SM powder sample was recorded with X-ray diffractometer (Bruker, D8, ADVANCE, Germany) with Ni-filtered CuK_α_ radiation (λ = 1.54 Å) operating in the reflection mode at a scan speed of 4°/min. Information about the functional group bonding between silver nanoparticles and the snail mucus matrix was obtained using the Fourier Transform Infra-Red (FTIR) spectroscopy (JASCO FT/IR-6100 FTIR spectrophotometer) recorded in the attenuated total reflection (ATR) mode with an ATR Pro One unit for the powder sample. Both for XRD and FTIR investigations, dried as-prepared powder samples were directly used without any further processing.

### Antimicrobial activity of silver nanoparticles in snail mucus matrix

The screening of antimicrobial activity of the resultant silver nanoparticles in mucus matrix was carried out by the agar well diffusion method using nutrient agar (NA) medium. The organisms studied in the present research namely *E. coli NCIM 2065, S. aureus NCIM 5021, K. pneumoniae NCIM 2957* and *P. aeruginosa ATCC 9027* were procured from the National Chemical Laboratory (NCL, Pune). The bacterial inocula were prepared from the colonies of 24 h old culture on nutrient agar medium. The inoculum was adjusted to final concentration of approximately 10^6^ CFU/ml for the bacteria. Silver nanoparticles-snail mucus dispersion (prepared by subjecting to ultrasonication for 10 min in sterile distilled water) was added in the wells of the test media which were previously inoculated with each test strain. Plates were incubated at 37 $^\circ$C and inhibition zones were measured after 24 h of incubation^62^. MIC (Minimum Inhibitory Concentration) represents the lowest concentration required to inhibit the growth of micro-organisms. It was characteristically determined by serially diluting silver nanoparticles in the concentrations of 2, 2.5, 3, 3.5, 4 and 4.5 µg/ml. Micro-organisms were grown in Mueller Hinton broth at 37 $^\circ$C. All assays were carried out for three times and the control test was performed with the mucus^63^. The MBC/MFC values of the silver nanoparticles in mucus matrix were determined by taking samples from tubes of the MIC assay which were subsequently sub-cultured on freshly prepared nutrient agar plates or potato dextrose agar plates, and incubated at 37 $^\circ$C or 28 $^\circ$C for 48 h, respectively. The MBC/MFC was taken as the concentration of silver nanoparticles that did not show any growth on a new set of agar plates^64^.

### Determining the growth curves of bacterial cells

To examine the growth curves, bacterial cell concentration in Muller-Hinton broth was adjusted to 10^6^ CFU/ml and exposed to silver nanoparticulate dispersion at different concentrations viz*.,*1/2 MIC, MIC and 2 MIC. Each culture was incubated in a shaking incubator at 37 $^\circ$C for 24 h. Growth curves of bacterial cell cultures were attained through repeated measurements of the optical density (OD) at 600 nm.

### Mode of action of silver nanoparticles on bacterial cells

MIC of silver nanoparticles was used to judge the mode of action on bacteria. For this purpose, the concentrations of bacteria were adjusted to 10^6^ CFU/ml and were exposed to silver nanoparticulate composites for 6 h. One ml of sample was withdrawn from each set and the concentration of reducing sugars, proteins, DNA and RNA was determined. The method of Heipieper was also followed to determine the leakage at UV260 and UV280 absorbing material^65^.

### Cytotoxicity assay

HeLa (cervical cancer) cell lines were obtained from the National Center for Cell Science (NCCS), Pune and maintained in T25 flasks with 10% (v/v) fetal bovine serum (FBS) containing Dulbecco’s Modified Eagle Medium (DMEM). Cells were maintained at 37 $^\circ$C under 5% CO_2_ and 95% air in a humidified atmosphere.

The suspensions of biologically synthesized silver nanoparticles—snail mucus composite were prepared by ultasonicating typical sample powder for 10 min in sterile DMSO (1%) and were subsequently tested for their cytotoxicity by using modified MTT [(3-(4,5-dimethylthiazol-2-yl)-2,5-diphenyltetrazolium bromide)] assay as described previously^66^. In brief, cells were seeded at the density of 1 × 10^5^ cells /ml in 96 well plates. The plates were incubated overnight in CO_2_ incubator (37 $^\circ$C under 5% CO_2_ and 95% air in a humidified atmosphere). Next day, cells were treated with synthesized nanoparticles at single concentration (10 µg/ml) and incubated for further 48 h. Paclitaxel was used as positive control. After incubation, cell medium was replaced with MTT (0.5 mg/mg) – Phosphate Buffered Saline (PBS) medium and incubated for 2 – 4 h to form the reduced MTT or Formazan crystals. This reduced MTT or Formazan crystals were solubilized by addition of 100 µl of SDS-DMF (sodium dodecyl sulfate—N, N-dimethylformamide) (20% SDS in 50% DMF). The optical density was read on a microplate reader (Spectramax plus 384 plate reader, Molecular Devices Inc) at 570 nm filter against blank prepared cell free wells. Absorbance given by the cells treated with the vehicle alone was taken as 100% cell growth. IC_50_ and MIC values were calculated from the graphs, using Origin Pro software. The percent cytotoxicity in the presence of test fractions was calculated by the following formula:

$$ {\text{Percent}}\;{\text{cytotoxicity}} = \left[ {\left( {{\text{average}}\;{\text{absorbance}}\;{\text{of}}\;{\text{control}}{-}{\text{absorbance}}\;{\text{of}}\;{\text{a}}\;{\text{compound}}} \right)/\left( {{\text{absorbance}}\;{\text{of}}\;{\text{control}}{-}{\text{absorbance}}\;{\text{of}}\;{\text{blank}}} \right)} \right] \times {\text{1}}00 $$

where DMSO treated cells formed the control and culture medium without cells was treated as blank.

## Conclusions

We have offered ecofriendly biogenic synthesis of silver nanoparticles involving unique biomaterial i. e. naturally secreted mucus of terrestrial snail *Achatina fulica*. Apart from utility of mucus in bio-reduction and bio-stabilization steps in the synthesis of AgNPs, the possibility of developing antimicrobial skin-care products has been the main motivational aspect of our research leveraging well-known antimicrobial activity of AgNPs and well-documented benefits of snail mucus in skin-care. Towards this end, we have ascertained antimicrobial performance of the resultant AgNPs-SM composite samples against different pathogens. Specifically, we have observed high antimicrobial activity against deadly pathogen *Pseudomonas aeruginosa* which tends to become resistant to various antibiotics in the hospital settings. Most importantly, in a pilot attempt, silver nanoparticles in biocompatible mucus matrix exhibited anti-cancer activity against HeLa (cervical cancer) cell lines. From the standpoint of future therapeutic applications based on our primary investigations, we put forward the possibility of realizing AGNPs-SM based topical cream/gel for effective acne treatment as well as for rapid wound healing without leaving scar on the affected skin. Such an idea can probably be extended to formulate anti-cancer cream/gel for specific topical application.

## Supplementary Information

Supplementary Information.
