# Supplementary material for: Terrestrial snail-mucus mediated green synthesis of silver nanoparticles and in vitro investigations on their antimicrobial and anticancer activities
Source: Sci Rep. 2021 Jun 22;11:13068. doi: 10.1038/s41598-021-92478-4 (PMC8219800; doi:10.1038/s41598-021-92478-4)
Supplement: Supplementary file 1 — Supplementary Information. [file 41598_2021_92478_MOESM1_ESM.docx]

**Supplementary Information**

**Terrestrial snail-mucus mediated green synthesis of silver nanoparticles and *in vitro* investigations on their antimicrobial and anticancer activities**

Pramod C. Mane^1^, Shabnam A. R. Sayyed^1^, Deepali D. Kadam^1^, Manish D.Shinde^2^, Amanullah Fatehmulla^3^, Abdullah M. Aldhafiri^3*^, Eman A. Alghamdi^3^, Dinesh P. Amalnerkar^4*^ and Ravindra D. Chaudhari^1*^


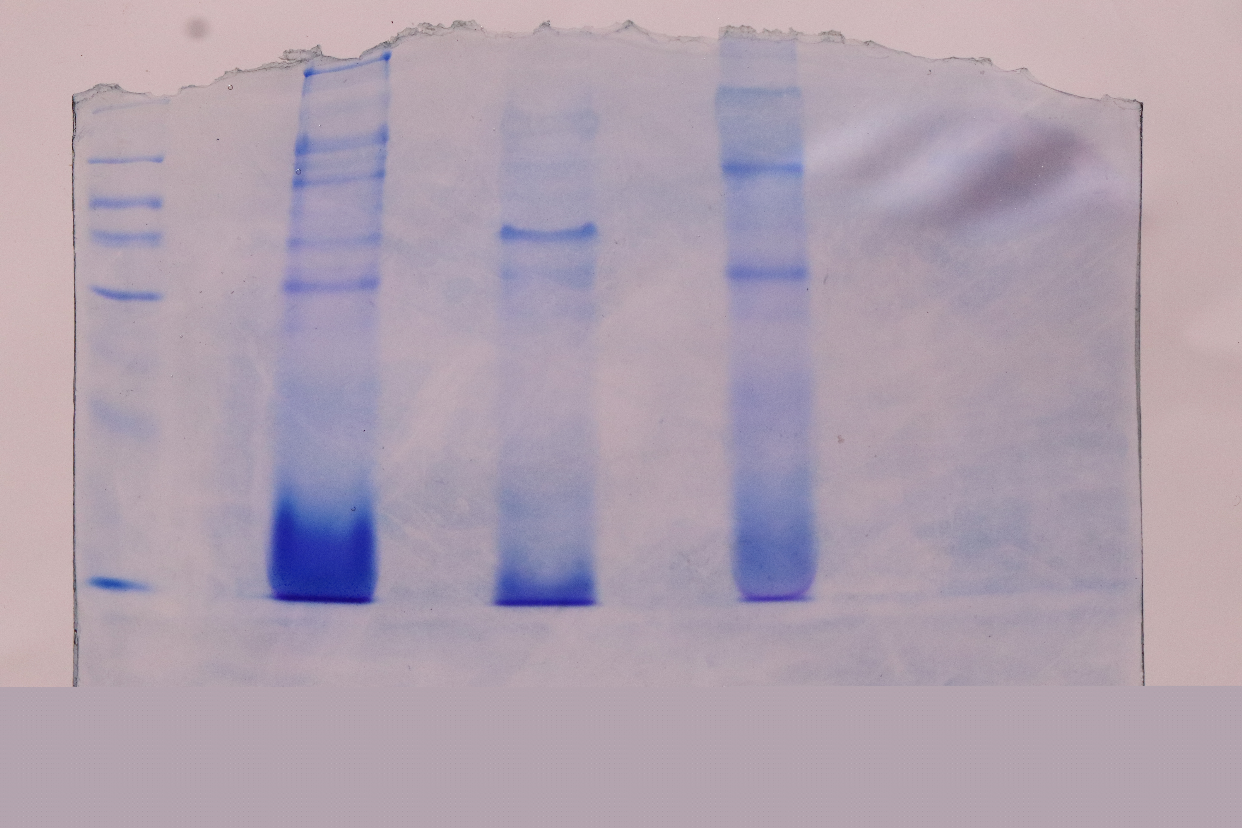


**Supplementary Figure S1.** Full-length image of electrophoretic pattern of standard protein marker as well as proteins of *A. fulica* mucus.


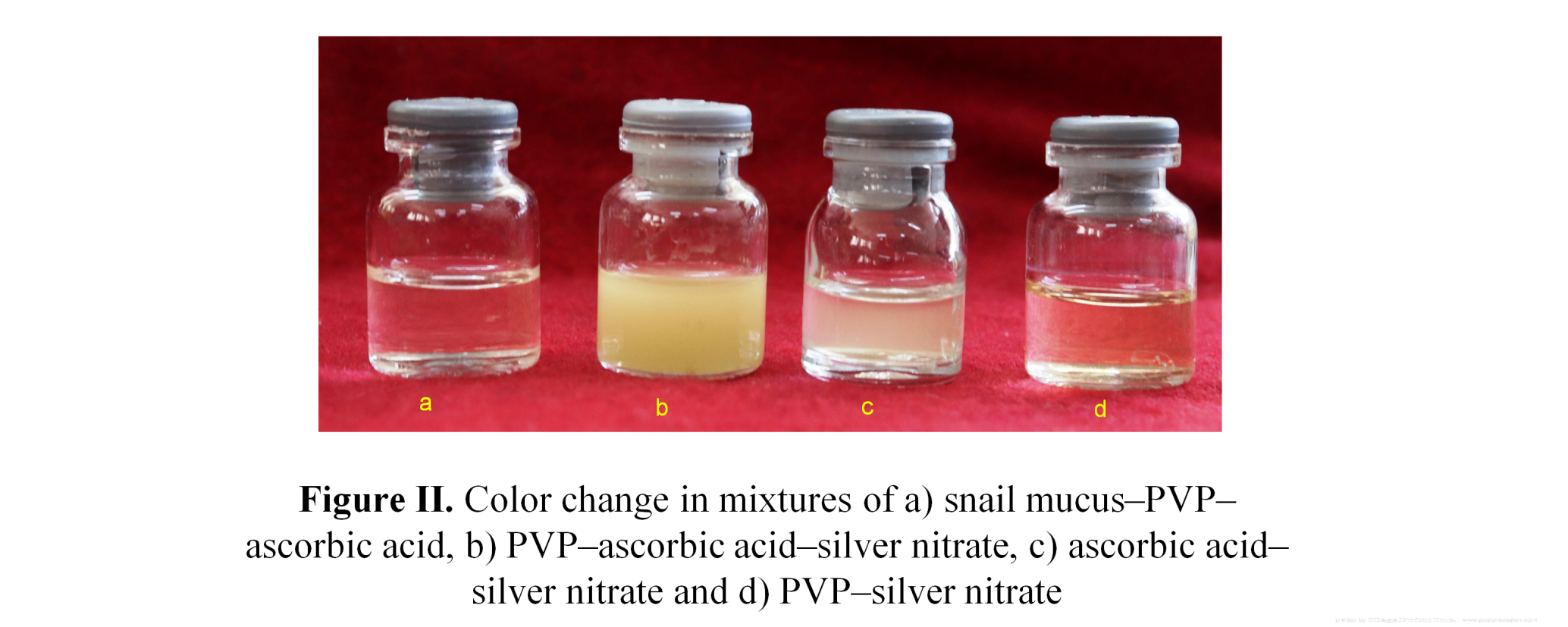


**Supplementary Figure S2.** Observed color change in admixtures corresponding to a) snail mucus–PVP–ascorbic acid, b) PVP–ascorbic acid–silver nitrate, c) ascorbic acid–silver nitrate and d) PVP–silver nitrate


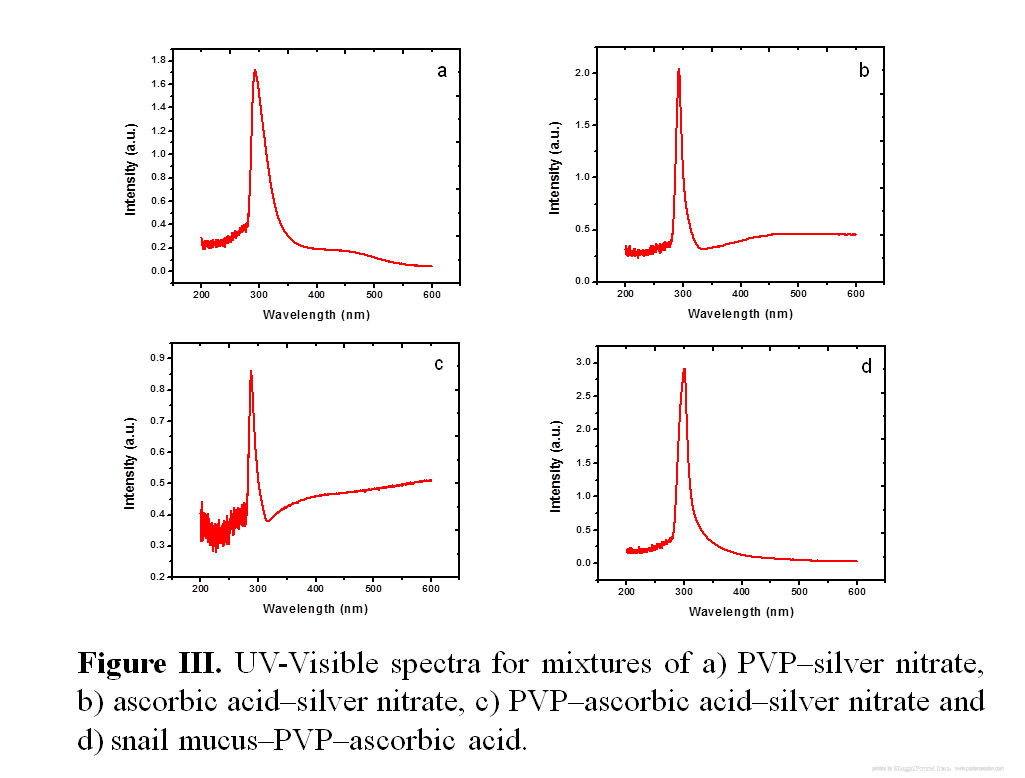


**Supplementary Figure S3.** UV-Visible spectra of admixtures of a) PVP–silver nitrate, b) ascorbic acid–silver nitrate, c) PVP–ascorbic acid–silver nitrate and d) snail mucus–PVP–ascorbic acid.


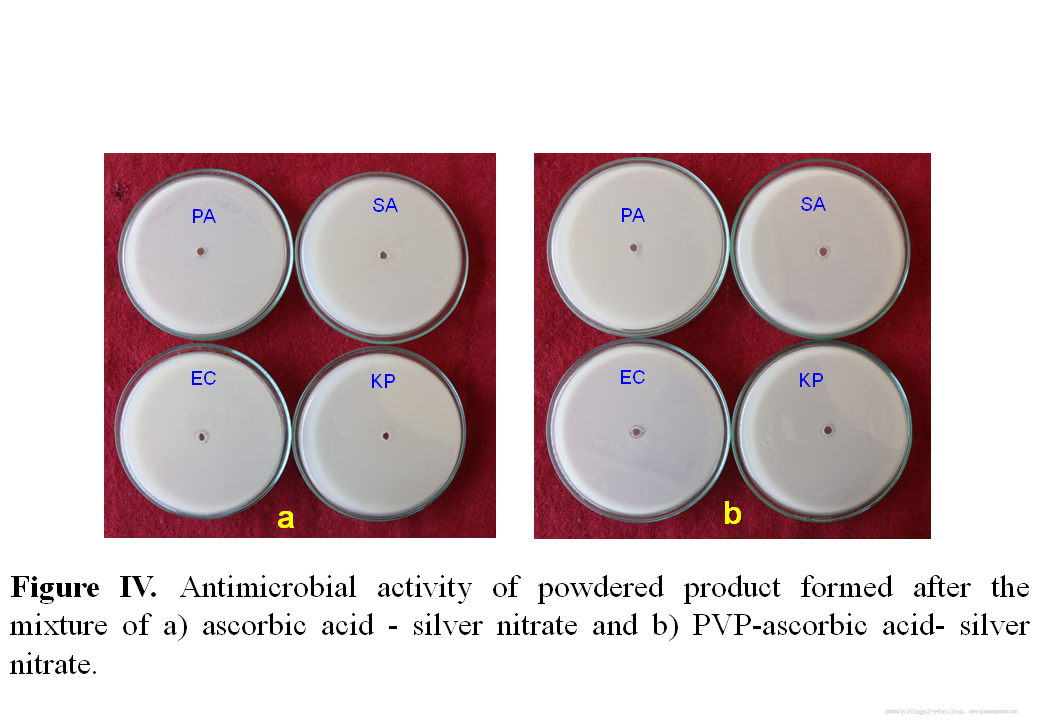


**Supplementary Figure S4.** Antimicrobial activity of powdered admixture product a) ascorbic acid - silver nitrate and b) PVP-ascorbic acid- silver nitrate.
